# Breast-feeding Protects against Arsenic Exposure in Bangladeshi Infants

**DOI:** 10.1289/ehp.11094

**Published:** 2008-03-06

**Authors:** Britta Fängström, Sophie Moore, Barbro Nermell, Linda Kuenstl, Walter Goessler, Margaretha Grandér, Iqbal Kabir, Brita Palm, Shams El Arifeen, Marie Vahter

**Affiliations:** 1 Institute of Environmental Medicine, Karolinska Institutet, Stockholm, Sweden; 2 MRC International Nutrition Group, London School of Hygiene and Tropical Medicine, London, United Kingdom, and MRC Keneba, The Gambia; 3 Institut für Chemie, Analytische Chemie, Karl-Franzens-Universität, Graz, Austria; 4 International Center for Diarrhoeal Disease Research, Bangladesh

**Keywords:** arsenic, blood, breast milk, drinking water, infants, saliva, urine

## Abstract

**Background:**

Chronic arsenic exposure causes a wide range of health effects, but little is known about critical windows of exposure. Arsenic readily crosses the placenta, but the few available data on postnatal exposure to arsenic via breast milk are not conclusive.

**Aim:**

Our goal was to assess the arsenic exposure through breast milk in Bangladeshi infants, living in an area with high prevalence of arsenic-rich tube-well water.

**Methods:**

We analyzed metabolites of inorganic arsenic in breast milk and infant urine at 3 months of age and compared them with detailed information on breast-feeding practices and maternal arsenic exposure, as measured by concentrations in blood, urine, and saliva.

**Results:**

Arsenic concentrations in breast-milk samples were low (median, 1 μg/kg; range, 0.25–19 μg/kg), despite high arsenic exposures via drinking water (10–1,100 μg/L in urine and 2–40 μg/L in red blood cells). Accordingly, the arsenic concentrations in urine of infants whose mothers reported exclusive breast-feeding were low (median, 1.1 μg/L; range, 0.3–29 μg/L), whereas concentrations for those whose mothers reported partial breast-feeding ranged from 0.4 to 1,520 μg/L (median 1.9 μg/L). The major part of arsenic in milk was inorganic. Still, the infants had a high fraction (median, 87%) of the dimethylated arsenic metabolite in urine. Arsenic in breast milk was associated with arsenic in maternal blood, urine, and saliva.

**Conclusion:**

Very little arsenic is excreted in breast milk, even in women with high exposure from drinking water. Thus, exclusive breast-feeding protects the infant from exposure to arsenic.

Drinking water may contain elevated concentrations of arsenic, a well documented potent toxicant and carcinogen [International Agency for Research on Cancer (IARC) 2004], leading to chronic, often lifelong exposure. Arsenic in the bedrock and mineral deposits easily dissolves into the surrounding ground-water, and enhanced levels are found in most countries. Globally, > 100 million people are exposed, and Bangladesh is one of the most severely affected countries. About 10 million tube wells were installed across Bangladesh in the last few decades to decrease the use of contaminated surface water as drinking water. Approximately 50% of the tube wells, providing water to about 57 million people in Bangladesh, contain arsenic at levels exceeding the World Health Organization (WHO) drinking-water guideline of 10 μg/L (British Geological Survey 2001; IARC 2004).

There is a wealth of data on arsenic-related health effects in adults, including various forms of cancer, skin lesions, diabetes mellitus, chronic bronchitis, cardiovascular disease, peripheral neuropathy, as well as hematologic, liver, and kidney effects (IARC 2004; National Research Council 2001). There is also increasing evidence of negative effects of exposure to arsenic in drinking water on fetal growth, fetal loss, and infant mortality (Hopenhayn-Rich et al. 2000; Huyck et al. 2007; Milton et al. 2005; Rahman et al. 2007; von Ehrenstein et al. 2006; Yang et al. 2003) as well as neurodevelopment in school-age children (Calderon et al. 2001; Dakeishi et al. 2006; Rosado et al. 2007; Tsai et al. 2003; Wasserman et al. 2007; Wasserman et al. 2004; von Ehrenstein et al. 2007), but little is known about the critical windows of exposure. Generally, the brain is particularly susceptible to toxic insult during development (Grandjean and Landrigan 2006). That may be particularly true for arsenic that readily crosses the placenta (Concha et al. 1998a) and exerts epigenetic effects via interactions with DNA methylation (Reichard et al. 2007) and interacts with multiple nuclear receptors (Bodwell et al. 2006). Such alterations may also cause functional changes via altered fetal programming, leading to increased susceptibility to diseases later in life (Heindel 2007; Vahter 2008). Thus, it is essential to assess exposure to arsenic early in life. However, the few available data on postnatal exposure to arsenic via breast milk are not conclusive. For example, indigenous women in the Argentine Andes exposed to about 200 μg/L arsenic in the drinking water showed very low excretion in breast milk (~ 3 μg/L) (Concha et al. 1998b), whereas a Bangladeshi study reported up to 38 μg/L in breast milk in a small group of seven women (Watanabe et al. 2003).

The aim of the present study was to assess the exposure to arsenic through breast milk in Bangladeshi infants who live in an area with elevated arsenic exposure because of arsenic in tube-well water (Vahter et al. 2006). We analyzed metabolites of inorganic arsenic (iAs) in breast milk and urine from 98 3-month-old infants, with detailed information on breast-feeding practices and maternal arsenic exposure, as measured by concentrations in blood, urine, and saliva. Arsenic is metabolized in the body by methylation, and the main metabolites produced—methylarsonic acid (MA) and dimethylarsinic acid (DMA)—are readily excreted in urine (Vahter 2002). Although an efficient methylation of arsenic to DMA increases the rate of excretion and is likely to reduce the health risks (Vahter 2002), the proportion of MA in urine has been associated with increased risk of a number of different health effects in adults (Tseng 2007), possibly because it is related to the intermediate trivalent and highly toxic MA (Bredfeldt et al. 2006) in the tissues (Vahter 2002).

## Materials and Methods

### Study area and individuals

The study was carried out in Matlab, about 53 km southeast of Dhaka, Bangladesh, where the International Center for Diarrhoeal Disease Research is running a health and demographic surveillance system recording all vital events, as well as a hospital and four health clinics. Our ongoing research project to evaluate effects of arsenic exposure via the drinking water on pregnancy outcome and child development is nested in a large randomized population-based food and micronutrient supplementation trial in pregnancy (the Maternal and Infant Nutrition Interventions of Matlab; MINIMat), which includes approximately 4,500 women recruited in early pregnancy (November 2001 to October 2003) and followed during pregnancy until 6 months postpartum. Urine and blood samples were collected repeatedly for evaluation of nutritional status and arsenic exposure, and breast milk samples were collected at 1, 2, 6, and 12 months postpartum. We previously reported that the pregnant women (subgroup recruited in 2002) showed a wide range of arsenic concentrations in their urine, largely reflecting the concentrations in the drinking water (Vahter et al. 2006).

An additional arm of the MINIMat study evaluated the effect of exclusive breast-feeding counseling on the rate and duration of exclusive breast-feeding. To validate the data on infant feeding practices reported by the women in questionnaires, a subcohort of 98 pairs of mothers and their 3-month-old infants was selected from August 2003 to March 2004 for assessment of breast-milk and non-breast-milk water intakes using the dose-to-mother deuterium dilution technique (Moore et al. 2007). Based on the measurement of deuterium in collected samples of maternal saliva and infant urine, infant intakes of breast-milk and non-breast-milk water were estimated. These data showed good agreement between actual intakes and those reported by questionnaire (Moore et al. 2007). We then used residual samples of infant urine and maternal saliva to evaluate the exposure to arsenic via breast milk. We analyzed the concentrations of arsenic metabolites in the infants’ urine samples and compared them with those in breast milk, collected from the mothers at about the same time. Breast milk samples were available for 79 (81%) of the mothers. Information on breast-feeding practices was obtained from the questionnaire concerning exclusivity of breast-feeding practices. The metabolite pattern in infant urine was compared with that in maternal urine, collected in early pregnancy [gestational week (GW) 8], before the pregnancy-related change in arsenic metabolism (Concha et al. 1998a).

**Figure 1:**
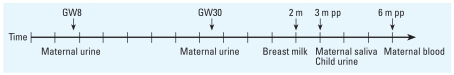
Sampling points, maternal urine GW8 and GW30, breast milk 2 months (m) postpartum (pp), maternal saliva and child urine at 3 m pp, and maternal blood 6 m pp.

To evaluate the excretion of arsenic in breast milk, we compared the milk arsenic concentrations with those in maternal saliva, collected at the same time as the infant urine samples. Because there is no previous information on the excretion of arsenic in saliva, we also measured arsenic concentrations in samples of maternal blood, collected 3 months later (6 months postpartum) from 38 (39%) of the mothers, and maternal urine, collected in late pregnancy (GW30; *n* = 91). Usually arsenic exposure via drinking water is chronic in nature, resulting in small variations in blood and urinary arsenic concentrations over time (Concha et al. 2002). However, we could not affirm that the maternal blood arsenic concentrations decreased over the 3–4 months between the collection of milk and the collection of blood and urine, because a parallel mitigation program screened the tube wells for arsenic, increased the awareness of arsenic in the tube wells, and worked to find alternative water sources for those with elevated arsenic concentrations (Jakariya et al. 2007; Rahman et al. 2006). To evaluate potential changes in maternal arsenic exposure, we also measured the concentrations of arsenic in blood samples collected from the studied mothers in early pregnancy (GW14). The different matrices and sampling points are shown in Figure 1.

### Sample collection

All samples of urine, blood, saliva, and breast milk were stored at –20°C or –70°C until transported frozen by air to freezers at the Karolinska Institute, and kept there until analysis. Maternal urine samples were collected as described previously for the women enrolled during January 2002–March 2003 (Vahter et al. 2006). Urine was collected from infants by placing cotton balls in disposable diapers and waiting for the infants to pass urine. The diaper was checked every 15 min and, if dry, clean cotton balls were inserted. If the infant had passed urine, the cotton balls were removed using forceps, and the urine was expressed using a plastic syringe. For the purpose of the deuterium dilution study, six urine samples were collected from all participating infants over a 15-day period (days 0, 1, 3, 4, 13, and 14). At the same time points, a saliva sample was collected from the mother of each infant, at least 30 min after consumption of any food or drink. Samples were collected by asking the mother to chew on a cotton ball for approximately 5 min, and then saliva was expressed using a plastic syringe. For the purpose of the current analysis, urine samples from each individual infant and saliva samples from each mother were pooled to provide a paired sample for each participating mother–infant pair. There was little arsenic (< 0.5 μg/L) released from the cotton ball when extracted with deionized water or urine.

In total, 79 of the studied mothers had collected breast milk 2–3 months (average, 9.5 weeks) postpartum when they visited the clinics for follow-up. Before milk collection, the breast was cleansed with water. Usually milk was collected from one breast while the other was given to the baby. The mother gently expressed 10 mL milk into a plastic tube. If sufficient milk was not expressed from one breast, the mother tried with the other breast. The samples were transported in cooling box to the Matlab hospital laboratory, where they were labeled and deep-frozen at –70°C.

Venous blood samples were collected in the health clinics using 5.5 mL Li-Heparin tube (Sarstedt, Nümbrecht, Germany), transported to the hospital laboratory for separation of erythrocytes, which were stored at –80°C, transported to Sweden, and analyzed for arsenic at the Karolinska Institute. Saliva was collected after 5 min of chewing on cotton to stimulate salivation (Moore et al. 2007).

### Measurements of arsenic concentrations

Measurements of total arsenic in maternal breast milk, saliva, and erythrocytes were performed by inductively coupled plasma mass spectrometry (ICPMS; Agilent 7500ce series) with an integrated sample introduction system (Agilent Technologies, Waldbronn, Germany). Before the ICPMS analysis, breast milk and erythrocytes were acid digested with 65% concentrated suprapur nitric acid (Merck, Darmstadt, Germany) in microwave-assisted autoclave (UltraClave; EMLS, Leutkirch, Germany). For quality control, reference material (Seronom Trace Elements Whole Blood L-1, Lot MR4206; L-2, Lot 0503109; SERO AS, Billingstad, Norway) with a reference value of 1.8 ± 0.40 μg/L and 13.2 ± 1.3 μg/L, respectively. We obtained an average at 2.2 μg/L and 14 μg/L, respectively (*n* = 17).

We measured the sum of iAs and the methylated metabolites, here referred to as urinary arsenic (U-As), in maternal urine by hydride generation–atomic absorption spectroscopy (HG-AAS) (Vahter et al. 2006). Separation of the different As metabolites [As(III), As(V), MA, and DMA] in urine was performed by a high performance liquid chromatography (HPLC) system (Agilent 1100 series system; Agilent Technologies), equipped with a Hamilton PRP-X100 anion-exchange column 4.1 × 250 mm (Reno, NV, USA) and coupled to hydride generation (HG) and ICPMS. HG is commonly coupled to the detector to discriminate for organic arsenic species like arsenobetaine in the urine, because these species do not form volatile arsines as iAs and its metabolites do. The method and the equipment have been described in detail elsewhere (Lindberg et al. 2007). For quality control, a reference urine (NIES CRM no. 18; National Institute for Environmental Studies, Ibaraki, Japan) with a certified DMA concentration of 36 ± 9 μg/L was analyzed together with the collected urine samples. The obtained average (± SD) DMA concentration was 37 ± 1.7 μg/L (*n* = 5). The correlation between measured U-As by HG-AAS and calculated U-As by adding the different metabolites measured by HPLC–HG–ICPMS was 0.98 (*n* = 87), demonstrating good column recovery.

To determine the arsenic compounds in breast milk, we added 10 μL of concentrated formic acid (Fluka, Buchs, Switzerland) to an aliquot of 500 μL breast milk to precipitate the proteins. Thereafter, the samples were centrifuged for 15 min at 15,000 rpm (Microliter centrifuge; Hettich, Tuttlingen, Germany) to separate fat, proteins, and whey. For the arsenic measurements, the fat layer was removed and the whey was carefully transferred into the polypropylene vials sealed with rubber caps (both from Agilent, Waldbronn, Germany). The arsenic compounds were determined with an HPLC system (Agilent 1100 series system; Agilent Technologies) coupled to an ICPMS (7500c; Agilent) equipped with a Babington type nebulizer. The metabolites were separated on a Hamilton PRP-X100 anion-exchange column, 4.1 × 250 mm with 20 mM aqueous ammonium phosphate (NH_4_H_2_PO_4_) solution at pH 6 [adjusted with ammonium hydroxide (NH_4_OH)] at a flow rate of 1.5 mL/min and a column temperature of 40°C. The injection volume was set to 20 μL. For signal enhancement, methanol (MeOH) was pumped to the ICPMS spray chamber as described elsewhere (Kovaãeviã and Goessler 2005). The signal was recorded at *m/z* 75 (^75^As) and *m/z* 77 (^40^Ar^37^Cl, ^77^Se). To evaluate the data, we used the ICPMS chromatographic software version C.01.00 (Agilent). The limit of detection was < 0.01 μg/L for both blood and saliva. For breast milk and urine it was 0.2 μg/L for As(V) and 0.1 μg/L for As(III), MA, and DMA.

To compensate for variations in urine dilution, we adjusted the arsenic concentrations to the average specific gravity (SG), measured by a digital refractometer (EUROMEX RD 712 clinical refractometer; EUROMEX, Arnhem, Holland). U-As was adjusted to the overall mean SG value of 1.003 g/mL in the infant urine and 1.012 g/mL in maternal urine, according to U-As × [(1.003 – 1) or (1.012 –1)]/(measured SG – 1). SG adjustment is shown to be less influenced by body size, age, and arsenic exposure than is creatinine adjustment (Nermell et al. 2008). The SG of saliva was about the same (1.003–1.004 g/mL) in all samples, so we did not adjust arsenic concentrations in saliva.

### Statistical methods

We used Spearman’s rank correlation analysis to evaluate the bivariate associations. We performed multiple regression analysis to evaluate the effect of the variables U-As, birth weight, and breast-feeding practice on the distribution arsenic metabolites. The analyses were performed with STATISICA 7.1 (Stat Soft, Inc., Tulsa, OK, USA). A *p*-value < 0.05 was considered statistically significant.

### Ethics

Approval for the MINMat study and the infant feeding validation study was obtained from the Ethical Review Committee, International Center for Diarrhoeal Disease Research, Dhaka, Bangladesh, and the Regional Ethical Committee at the Karolinska Institute. Written informed consent was obtained from all participating mothers.

## Results

The studied infants were on average 14 weeks old and had a mean body weight of 5.6 kg. More detailed demographic data are given in Table 1. The median arsenic concentration in infant urine, expressed as the sum of the arsenic metabolites (iAs, MA, and DMA) and adjusted to the average SG (1.003 g/mL), was 1.2 μg/L (Table 2). The distribution was skewed with a mean value of 23 μg/L. There were three urine samples with > 100 times the median concentration—142, 324, and 1,517 μg/L—whereas the highest among the others was 29 μg/L. The arsenic concentrations in urine were significantly lower in the infants that were exclusively breast-fed (EBF) (median U-As, 1.1 μg/L; 10th–90th percentiles, 0.27–6.7 μg/L, maximum 29 μg/L), than in those that were nonexclusively breast-fed (NEBF, i.e., predominantly or partly breast-fed) (median U-As, 1.9 μg/L; 10th–90th percentiles, 0.36–140 μg/L, maximum 1,520 μg/L; *p* < 0.05).

**Figure 2:**
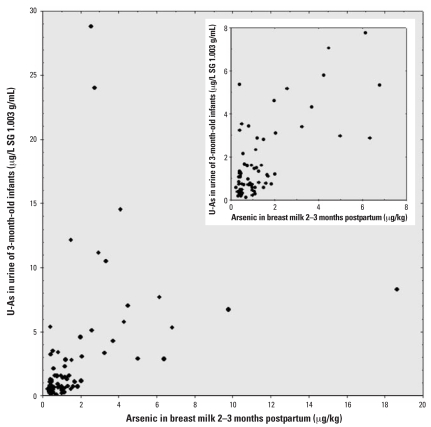
Scatterplot between the sum of arsenic metabolites (iAs, MA, and DMA) in urine (μg/L, SG 1.003 g/mL) of 3-month-old infants and the concentrations of arsenic in breast milk (μg/kg) (*r*_s_ = 0.64, *p* < 0.001, *n* = 79). Inset shows an enlargement of the diagram at the low concentration range.

The median arsenic concentration in breast milk was 1.0 μg/kg ranging up to 19 μg/kg (Table 2). There was a significant association between arsenic in infant urine and breast milk (*r*_s_ = 0.64, *p* < 0.001, *n* = 79; Figure 2), although there were some infants with high concentrations in urine in spite of low concentrations in breast milk. The arsenic in the breast milk samples was essentially in the form of iAs, and mainly As(III). As(V) was above the detection limit (0.2 μg/kg) in 11 of 79 samples, whereas DMA could be detected in 38 and MA in 19 breast milk samples. Generally the arsenic metabolite pattern was concentration dependent: At low arsenic concentrations (≤ 1μg As/L) only As(III) was observed; at higher arsenic concentrations, As(III), DMA, MA, and As(V) were found, but even then the major fraction was iAs.

As shown in Table 3, the infant urine contained 8.2% iAs, 2.8% MA, and 87% DMA (median values). There was no significant difference in the distribution of the arsenic metabolites by sex or socioeconomic status, but both percent (%)DMA (negatively) and %iAs (positively) were associated with U-As and birth weight, whereas %MA was associated (negatively) with U-As and breast-feeding (*p* < 0.05). Using multivariate analysis, with one model for each arsenic metabolite, we tested whether the distribution was associated with U-As, birth weight, and breast-feeding. After excluding the outlier (U-As, 1,517 μg/L), the models showed that %DMA was negatively associated with birth weight (β = –0.23, *p* < 0.05) and that %MA was positively associated with NEBF and birth weight (β = 0.31 and 0.25, respectively; *p* < 0.05). No association was seen for %iAs. The %DMA and %MA in urine were not associated with current body weight or weight gain since birth. The highest urinary arsenic concentration (1,517 μg/L, excluded in the multivariate model) had 32% MA and only 15% DMA. There was no significant association between %MA or %DMA in infant and maternal (GW 8) urine.

**Figure 3:**
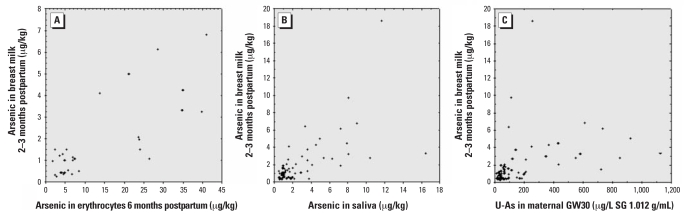
Scatterplots between arsenic in breast milk (2–3 months postpartum) against (*A*) arsenic in erythrocytes, 6 months postpartum (*r*_s_ = 0.71 *p* < 0.001, *n* = 31); (*B*) arsenic in saliva (μg/kg; 3 months postpartum (*r*_s_ = 0.61, *p* < 0.001, *n* = 79); and (*C*) the sum of arsenic metabolites (iAs, MA, and DMA) in maternal urine GW30 (SG 1.012 g/mL) (*r*_s_ = 0.64, *p* < 0.001, *n* = 76).

We estimated maternal exposure by arsenic concentrations in urine (GW30), saliva (3 months postpartum), and erythrocytes (6 months postpartum). The arsenic concentrations in saliva were low (median, ~1 μg/kg), whereas blood and urine contained median arsenic concentrations at 5.7 and 67 μg/L, respectively, with rather wide variations (Table 2). Maternal U-As concentrations were adjusted to the average SG 1.012 g/mL. As shown in Figure 3, there were significant correlations between arsenic concentrations in breast milk and all other maternal biomarkers of exposure: erythrocytes (*r*_s_ = 0.71, *n* = 31; *p* < 0.001), saliva (*r*_s_ = 0.69; *n* = 79; *p* < 0.001), and urine (*r*_s_ = 0.61, *n* = 79; *p* < 0.001). The correlations between arsenic concentrations in all the maternal biomarkers are shown in Table 4.

## Discussion

The present study is the first to clarify that the excretion of arsenic in breast milk is mainly in the trivalent inorganic form (arsenite) and that there is a significant association between arsenic concentrations in milk and maternal blood. Still, arsenic concentrations in breast milk were generally low in the studied rural Bangladeshi women (about 1 μg/kg), despite high arsenic exposures. Consequently, the arsenic concentrations in urine from the EBF infants were low, mostly around 1 μg/L. This finding confirms previous reports on low transfer of arsenic to breast milk in lactating mothers in other countries (Concha et al. 1998b; Samanta et al. 2007).

Although the 90th percentile of the arsenic concentrations in urine of EBF infants was 6.7 μg/L, or about 10% of that in maternal urine, two infants had concentrations > 20 μg/L. Whether this originated from breast milk or the child had been given some water or semi-solid food is not known. Because the breast milk samples in general were collected a few weeks before the collection of infant urine, the mothers might have had an occasional very high intake of arsenic at the time of the infant urine collection. Also, we cannot entirely rule out the possibility that the samples had been contaminated by arsenic—for example, from water used for cleansing the infant—although this would likely have a minor influence on the concentrations (e.g., the addition of 0.1 mL of water with 500 μg As/L to 10 mL urine would only increase the concentrations by 5 μg/L). The children with the very highest arsenic concentrations in urine (142, 324, and 1,517 μg/L), were not exclusively breast-fed, but were receiving other, complementary foods (water, other liquids, semisolid foods), in addition to breast milk.

We show for the first time that human breast milk contains almost entirely iAs, mainly in the trivalent form, which implies that the infants are exposed mainly to arsenite via breast milk, although at low doses. As(V) was detected in 14% of the samples, but we cannot exclude that some As(III) had been oxidized after sampling, during transport and storage of the milk samples. The results indicate that the methylated arsenic metabolites in blood plasma do not easily pass over the mammary glands. Indeed, we found that the arsenic concentrations in breast milk were negatively correlated with %DMA (*r*_s_ = –0.19) and positively correlated with %iAs (*r*_s_ = 0.16) in maternal urine. Thus, an efficient maternal methylation of iAs leads to less arsenic excretion in breast milk. Possibly, As(III), which is the only arsenic metabolite that is protonated at physiologic pH (pK_a1_ of arsenous acid = 9.2), passes via aquaglyceroporins, which are the main transporters of As(III) in most organisms (Liu et al. 2004; Rosen 2002), and present in the mammary gland during lactation (Matsuzaki et al. 2005). Arsenate is likely to be transported by the phosphate transporters (Rosen 2002). Assuming somewhat lower arsenic concentrations in plasma compared with erythrocytes (WHO 2001) and a similar pattern of arsenic metabolites in blood plasma as in urine at about 20% iAs (Gamble et al. 2007), the results indicate a breast milk:plasma ratio of about one for iAs. This implies no barrier towards iAs in the mammary gland.

The present study is also the first to demonstrate an efficient methylation of iAs in infants. We observed very high fractions of DMA in the infant urine (overall median of 87% and 89% in EBF infants). The average %MA in urine, which is known to be associated with increased health risks in adults [for review, see, e.g., Lindberg et al. (2008); Tseng (2007)], was only 2.8%, (2.3% in EBF infants) compared with 10% in the studied mothers and 10–20% in most studied population groups (Vahter 2002). Similar high %DMA and even lower %MA were reported for newborn babies in the Andes, Argentina, which was related to the efficient arsenic methylation in late pregnancy, resulting in transfer of mainly DMA to the fetus before birth (Concha et al. 1998a). We recently proposed (Vahter 2007) that this high level of arsenic methylation in pregnant women is a result of the *de novo* synthesis of choline by phosphatidyle-tanolamine methyltransferase, which is up-regulated in pregnancy to meet the fetal demand of choline for brain development (Zeisel 2006b). The endogenously produced choline, via betaine and betainehomocysteine methyl-transferase, is also the only alternate pathway to the folate-linked remethylation of homocysteine to methionine, and therefore particularly important in women with low intake of folate (Velzing-Aarts et al. 2005; Zeisel 2006a), such as those in the present study (Li et al. 2007). An efficient removal of homocysteine is a prerequisite for an efficient one-carbon metabolism, by which arsenic is methylated. The endogenous maternal synthesis of choline provides a high level of circulating free choline in the newborn child (Ilcol et al. 2005), which may support the methylation of ingested arsenic. In addition, the folate concentration in the fetal liver is nearly 50% higher than in the maternal liver, and contributes to an appropriate metabolic activity in the developing child (Maloney et al. 2007; Wallace et al. 2008). Folate in breast milk reaches maximum levels first some months postpartum (Khambalia et al. 2006; Monsen et al. 2003), whereas appreciable amounts of choline are present already in the first mature milk (Holmes et al. 2000; Ilcol et al. 2005) to meet the demand of choline for the developing brain, as well as a continuous adequate one-carbon metabolism and methylation of arsenic, as shown in the present study. The choline content of human breast milk doubles during the first weeks after delivery (Holmes et al. 2000), by active uptake from maternal circulation and *de novo* synthesis from phosphatidylethanolamine in the mammary gland (Yang et al. 1988).

Unexpectedly, birth weight, but not current body weight or weight gain, was positively associated with infant urinary %MA and negatively associated with %DMA. The reason for this is not known, but possibly it is related to elevated infant homocysteine levels, a known risk factor for elevated %MA in urine (Gamble et al. 2005). Unlike the situation in older children and adults, low homocysteine levels in infants are critically dependent on adequate vitamin B_12_ levels, which are related to the maternal vitamin B_12_ status (Ueland and Monsen 2003). There seems to be insufficient vitamin B_12_ transfer over the placenta, leading to low newborn stores, particularly in mothers with low vitamin B_12_ status. This was the case for many of the Matlab women (Li et al. 2007) as well as many other poor women, especially with diets low in meat (Allen 2005; Specker et al. 1990). In addition, a low serum vitamin B_12_ level in the mother results in low concentration in breast milk, and still lower vitamin status and higher homocysteine in the infant. Probably, high-birth-weight infants have depleted the maternal vitamin B_12_ stores to a larger extent than small infants, both during the growth spurt in late gestation and post-natally, as they consume more breast milk.

The extent to which the observed efficient arsenic methylation protects against toxic effects of arsenic exposure during fetal life and infancy remains to be elucidated. There was a clear tendency of increasing %MA with increasing exposure (increasing U-As). The two infants with U-As concentrations at 140 and 220 μg/L had 6–9% MA and the infant with > 1,500 μg/L had 32% MA in the urine. This is most likely attributable to the inhibition of the arsenic methyl transferase by the excess iAs. It has previously been shown that the second methylation step is sensitive to high concentrations of arsenic (Li et al. 2007; Lindberg et al. 2008).

This is the first report on arsenic concentrations in saliva in relation to blood arsenic. We can conclude that the concentrations were very low, at a similar level as in breast milk and about 20% of those in erythrocytes. Still, the saliva samples might have been slightly contaminated by arsenic from the cotton used during the sampling. Although there was a significant correlation between arsenic in saliva and blood (*r*_s_ = 0.63), the low concentrations of arsenic and the small variation make salivary arsenic unsuitable as a biomarker of exposure. Interestingly, Yuan et al. (2008) reported much higher concentrations of arsenic in saliva from Inner Mongolia, China, and the metabolite pattern was similar to that in drinking water: essentially iAs.

The main strengths of this study include the carefully collected urine from the infants (six urine samples over 2 weeks from each child), the validated data on breast-feeding practices, and the arsenic measurements, using the highly sensitive ICPMS method, coupled to HPLC–HG generation to enable speciation of the arsenic metabolites as measure of arsenic methylation efficiency in the infants. The main disadvantages are the collection of maternal samples at different points in time compared with the collection of infant urine. However, we believe that the blood arsenic concentrations in the mothers did not change much over time. The arsenic concentrations in erythrocytes collected 6 months postpartum (mean, 16 μg/kg) were about the same as that in blood collected in early pregnancy (13 μg/L, paired samples), which would speak against a change to water sources with lower arsenic concentrations in response to the mitigation activities. Similarly, the median arsenic concentration in maternal urine in GW30 was similar to that in the urine collected in early pregnancy. Thus, we can conclude that the blood collected 3–4 months after the breast milk samples and the urine collected 4.5 months before the milk samples represented the maternal exposure at the time of the infant study. Our previous study showed an overall median concentration of 80 μg/L in the urine of women (*n* = 1,944) in Matlab during 2002 and first months of 2003 (Vahter et al. 2006). However, the women in the geographic area B, where the main hospital is located and where the women in the present study lived, had 55 μg/L in their urine, similar to levels in the women included in the present study.

In conclusion, we have shown that very little arsenic, mainly as iAs, is excreted in human breast milk, even in women with high exposure to arsenic via drinking water. Also, we found an efficient methylation of the ingested iAs in the infants. This information is important from a public health point of view because it implies that the breast-fed infants, particularly those who are exclusively breast-fed, are protected from exposure to arsenic during this critical period of development. However, to what extent breast-feeding decreases the health risks associated with pre-natal arsenic exposure remains to be elucidated. There is an urgent need for evaluation of arsenic in infant formula and weaning food in arsenic-contaminated areas.

## Correction

In Table 4, the *p*-value is 0.001, not 0.5 as in the manuscript originally published online, and has been corrected here.

## Figures and Tables

**Table 1:** Demographic data on the participating children (*n* = 98), with 47% girls and 52% boys.

Characteristic	Value	Min–Max
Age (weeks)	14 ± 1.4	11–17
Weight (kg)	5.6 ± 0.76	3.3–7.4
Birth weight (g)	2,733 ± 387	1,480–3,553
Gestational week at birth	39 ± 1.8	30–43
Parity	1.6 ± 1.6	0–7
SES [wealth index (%)]^a^		
1	18	
2	28	
3	17	
4	14	
5	22	

Abbreviations: Max, maximum; Min, minimum. Values are mean ± SD or percent.

a Wealth index was based on information on household assets and estimated by principal component analysis, producing a weighted score.

**Table 2:** Concentrations of arsenic in infant urine at 3 month of age, breast milk, saliva, and blood, as well as maternal urine (GW8 and GW30).

	No.	Mean	Median	10th–90th	Min–Max
Infant urine, 3 months (μg/L)^a^	98	23	1.2	0.27–8.3	0.10–1,520
Breast milk, 2–3 months pp (μg/kg)	79	1.8	1.0	0.38–4.3	0.25–19
Saliva, 3 months pp (μg/kg)	98	2.5	1.3	0.69–6.3	0.51–17
For evaluation of changes over time					
Erythrocytes, GW14 (μg/kg)	13	13	9.2	2.6–28	2.3–39
Erythrocytes, 6 months pp (μg/kg)	38	13	5.7	2.8–35	1.8–41
Urine, GW8 (μg/L)^b^	97	142	49	22–434	12–810
Urine, GW30 (μg/L)^b^	91	167	67	21–550	10–1,130

Abbreviations: Max, maximum; Min, minimum; pp, postpartum. 10th and 90th are percentiles.

a Adjusted to average SG of 1.003 g/mL.

b Adjusted to average SG of 1.012 g/mL.

**Table 3:** Distribution of arsenic metabolites in infant urine at 3 months of age (*n* = 98) and maternal urine at GW8 (*n* = 97).

	As μg/L	%iAs	%MA	%DMA
All infants^a^				
Median	1.2	8.2	2.8	87
Mean	23	12	3.8	84
10th–90th	0.27–8.3	0–29	0–8.9	68–100
Range	0.10–1,520	0–60	0–32	15–100
Maternal GW8^b^				
Median	49	14	9.5	77
Mean	142	14	9.8	76
10th–90th	22–430	4.5–24	4.7–16	66–85
Range	12–810	0–39	3.1–22	56–97

Values represent micrograms per liter (% of total arsenic metabolites). 10th and 90th are percentiles.

a Infant U-As was adjusted to SG 1.003 g/mL.

b Maternal U-As was adjusted to SG 1.012 g/mL.

**Table 4:** Correlation between arsenic concentrations in all the maternal biomarkers, as evaluated by Spearman rank order correlation test.

	Breast milk	Saliva	Erythrocytes (6 months pp)	U-As GW8	U-As GW30
Breast milk		0.61	0.71	0.48	0.64
Saliva	0.61		0.63	0.56	0.61
Erythrocytes (6 months pp)	0.71	0.63		0.53	0.53
U-As GW8	0.51	0.56	0.63		0.59
U-As GW30	0.64	0.61	0.53	0.59	

pp, postpartum.

All correlations are statistically significant (*p* < 0.001).
